# Occult fractures of the proximal femur: imaging diagnosis and management of 82 cases in a regional trauma center

**DOI:** 10.1186/s13017-015-0049-y

**Published:** 2015-11-18

**Authors:** Bogdan Deleanu, Radu Prejbeanu, Eleftherios Tsiridis, Dinu Vermesan, Dan Crisan, Horia Haragus, Vlad Predescu, Florin Birsasteanu

**Affiliations:** I-st Clinic of Orthopedics and Trauma, Pius Brinzeu Emergency Clinical County Hospital, 10 I. Bulbuca Blvd, 300737 Timisoara, Romania; Victor Babes University of Medicine and Pharmacy, 2 Eftimie Murgu Square, 300041 Timişoara, Romania; Department of Radiology, Pius Brinzeu Emergency Clinical County Hospital, 10 I. Bulbuca Blvd, 300737 Timisoara, Romania; St. Pantelimon Clinical Emergency Hospital, 340 - 342 Pantelimon Road, Sector 2, 033092 Bucharest, Romania; Carol Davila University of Medicine and Pharmacy, 8 Eroii Sanitari Blvd, 050474 Bucharest, Romania; Aristotle University Medical School, 54124 Thessaloniki, Greece

**Keywords:** Occult, Hip, Fracture, X-ray, MRI, CT scan

## Abstract

**Background:**

Occult hip fractures are often difficult to identify in busy trauma units. We aimed to present our institutions experience in the diagnosis and treatment of occult fractures around the hip and to help define a clinical and radiological management algorithm.

**Method:**

We conducted a seven-year retrospective hospital medical record analysis. The electronic database was searched for ICD-10 CM codes S72.0 and S72.1 used for proximal femoral fractures upon patient discharge. We identified 34 (4.83 %) femoral neck fractures and 48 (4.42 %) trochanteric fractures labeled as occult.

**Results:**

The majority of the cases were diagnosed by primary MRI scan (57.4 %) and 12 were diagnosed by emergency CT scan (14.6 %). For the remaining cases the final diagnosis was confirmed by 72 h CT scan in 9 patients (representing 39 % of the false negative cases) or by MRI in the rest of 14 patients. MRI was best at detecting incomplete pertrochanteric fracture patterns (13.45 % of total) and incomplete fractures of the greater trochanter (3.65 % of total) respectively. It also detected the majority of Garden I femoral neck fractures (20.7 % of total). CT scanning accurately detected 100 % of Garden 2 fractures (2.44 %) and 25 % (3.65 %) of the complete pertrochanteric fractures (false negative 25 %).

**Conclusion:**

Occult fractures should be suspected in all patients with traumatic onset of hip pain that is inconsistent with normal radiographic findings. MRI is the golden standard but not as readily available not as cheap and not quite as quick to perform as as a CT scan. The latter which in turn can provide falsely negative results in the first 24 h. Improved imaging protocols could expedite management and improve treatment.

## Background

The estimated prevalence of occult hip fractures varies between 2 and 10 % of the total hip fractures [1–7].

Occult are defined, those fractures that cannot be detected by radiographic standard examination until several weeks after injury [8]. Their importance resides in their “occult” status, meaning that they are not identifiable by routine emergency X-rays. In the Emergency Room (ER) most of the times only a pelvic or hip AP view is obtained, with some services requiring a complete trauma X-Ray set with lateral, inlet, outlet and Judet oblique views. The necessity of these explorations is however disputed as the presence of a concomitant pelvic fracture either radiographically visible or occult and a occult proximal femur fracture has been excluded by previous studies [9]. Radiographically unapparent fractures can be easily mislabeled as hip soft tissue trauma and treated conservatively, with no restriction on weightbearing.

Definitive diagnosis of occult fractures of the proximal femur is without exception an imaging one. MRI is the imaging method of choice, better tolerated by patients providing a faster diagnosis of occult fractures of the hip, it is therefore recommended that the MRI is performed in an emergency setting whenever it is available [2, 4, 5, 10]. But not all services can provide emergency access to MR scanning, while CT scanning is more widespread and cheaper to perform. There is still no consensus over the use of CT in detecting occult hip fractures and it has been demonstrated that even modern 64 slice machines can yield false negative results [11].

We aimed to present our institutions experience in the diagnosis and treatment of occult fractures around the hip and to help defining a clinical and radiological management algorithm.

## Method

**Fig. 1:**
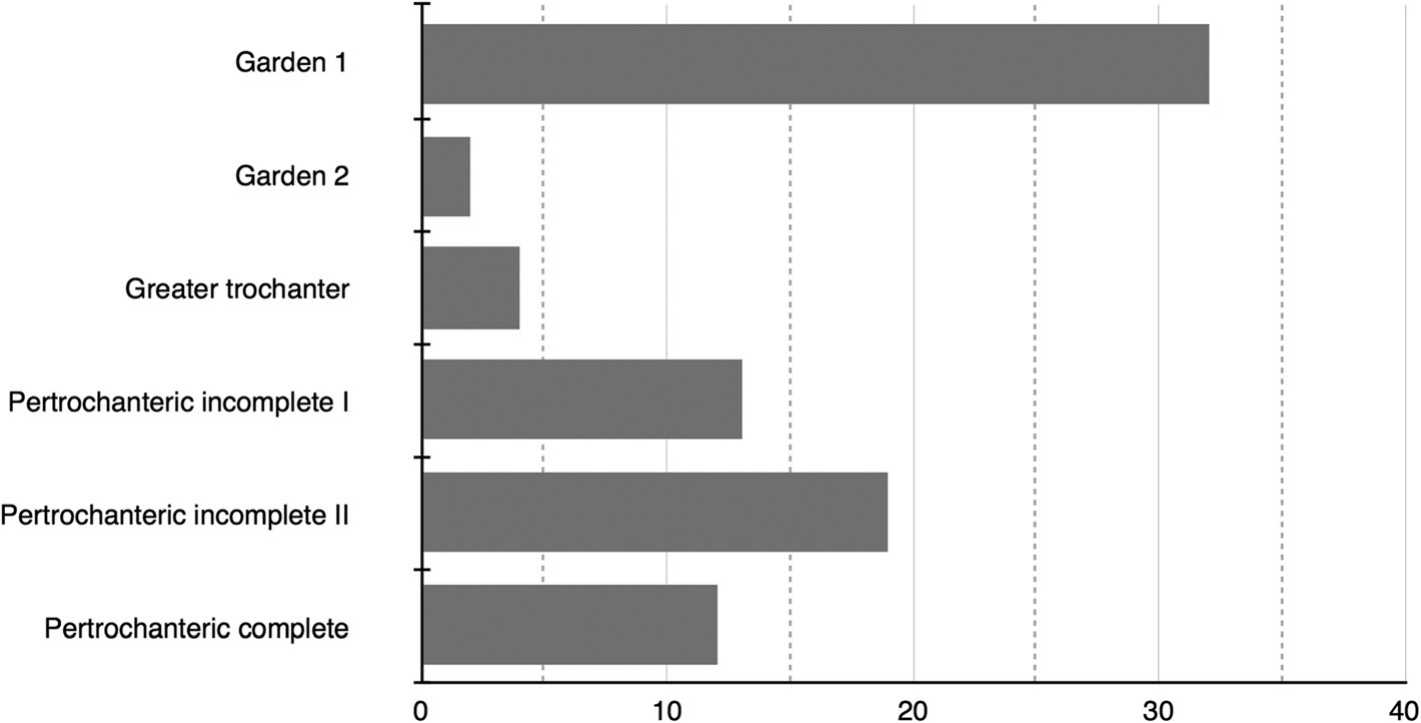
Distribution of the occult fractures in fracture types. Pertrochanteric incomplete I represents <50 % of the bicortical distance fractured. Pertrochanteric incomplete II represents >50 % of the bicortical distance fractured

We conducted a seven-year retrospective search on the hospital medical record (Hipocrate, RCS Software, Bucharest, Romania) between 2005-2012. The electronic database of a regional trauma center was searched for ICD-10 codes S72.0 (intracapsular) and S72.1 (extracapsular) used for proximal femoral fractures upon patient discharge. We identified 769 femoral neck and 926 pertrochanteric fractures that were treated in our clinic during this period. Out of these, 34 (4.83 %) femoral neck fractures and 48 (4.42 %) pertrochanteric fractures were labeled as occult. The fracture distribution by fracture subtype can be seen in Fig. 1.

Emergency AP radiographs routinely investigate hip trauma with the hips in neutral rotation. These are almost always sufficient for establishing a diagnosis and a management strategy for the case. For cases with suspected occult fracture further imaging was carried out either with an MRI (General Electric Signa Horizon LX 1 Tesla) or a CT scan (Philips Brilliance MX 16 slice). No contrast was used in either method. For the cases with an inconclusive emergency CT and eloquent hip symptoms such as pain in the Scarpa triangle, in the greater trochanter region, on weight-bearing or during passive movement of the hip joint, an MRI scan was performed during the following 24 h or a second CT scan at 72 h for establishing the diagnosis.

Five patients were self-discharged from the ER without allowing us to establish final diagnosis. All five patients were reexamined in the next 1-3 days by a senior orthopedic surgeon due to ongoing hip symptoms and an outpatient MRI scan was performed. Most patients with hip fractures are elderly patients and the occult fractures group fitted this demographic distribution with 80 patients that were aged 60 and over, out of which 67 were aged 70 or over and only two young patients who were aged less than 60 years. The patients that decided to leave the ER were all over 75 years of age and did so upon consultations with their families. Thirty-eight patients had at least one neurological condition such as Parkinson’s disease, cortical atrophy or stroke history, which ranged in severity from mild to serious. The majority of diagnosed cases were treated surgically whilst conservative management was reserved for the incomplete and totally undisplaced occult fractures according to the literature [12, 13].

Suspicion of an occult hip fracture should arise when there is a inconsistency between the trauma history and physical examination on one hand and the imaging results on the other. For example a young patient with high-energy hip trauma that has a negative X-ray but presents with a nonweightbearing painful hip should be further investigated. The patient may be able to bear weight but also present significant hip pain spontaneous and/or on palpation mobilization and this should also prompt for further imaging studies. Elderly patients with occult hip fractures almost always have sustained a trivial trauma and present non or partial weightbearing with pain in the anterior portion of the hip.

All data were recorded electronically using Microsoft Office Excel spread sheets (Microsoft Corporation, Redmond, US-WA) and the statistical analysis was performed using XLStat (Addinsoft SARL New York, US-NY). Approval for this study was obtained from our Hospital’s institutional review board (IRB) and informed consent was obtained from each patient.

## Results

**Fig. 2:**
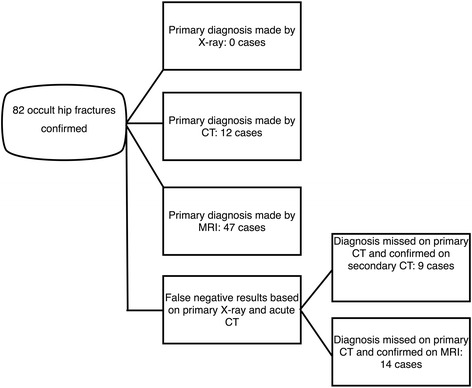
Case stratification showing the diagnostic algorithm used and the number of fractures diagnosed primarily and secondarily by MRI and CT scan

From a total of 82 identified occult fractures 47 were diagnosed by MRI (57,4 % 95 % CI: 27,4 %–90,9 %) and 12 were diagnosed by emergency CT (14,6 % 95 % CI: 6,0 %-21,9 %). For the remaining 23 cases the final occult fracture diagnosis was confirmed by 72 h CT scan in 9 cases (11 % 95 % CI: 3,7 % – 9,5 % representing 39 % of the false negative cases) or by MRI in the rest of 14 cases (17 % 95 % CI: 3 % – 33,6 %, representing 61 % of the false negative cases) (Fig. 2).

MRI was best (*p* = 0.048) at detecting incomplete pertrochanteric fracture patterns in 84,6 % of the cases (13,45 % of total 95%CI: 12,1 % – 20,7 %) and incomplete fractures of the greater trochanter 75 % (3,65 %) and detected the majority (53 %) of Garden I femoral neck fractures (20,7 % of total). CT scanning accurately detected 100 % of Garden 2 fractures (2,44 % of total) and 25 % of the complete pertrochanteric fractures (3,65 % of total) but with more false negative results (23 false negative results based on acute scan; 28 % of total).

**Fig. 3:**
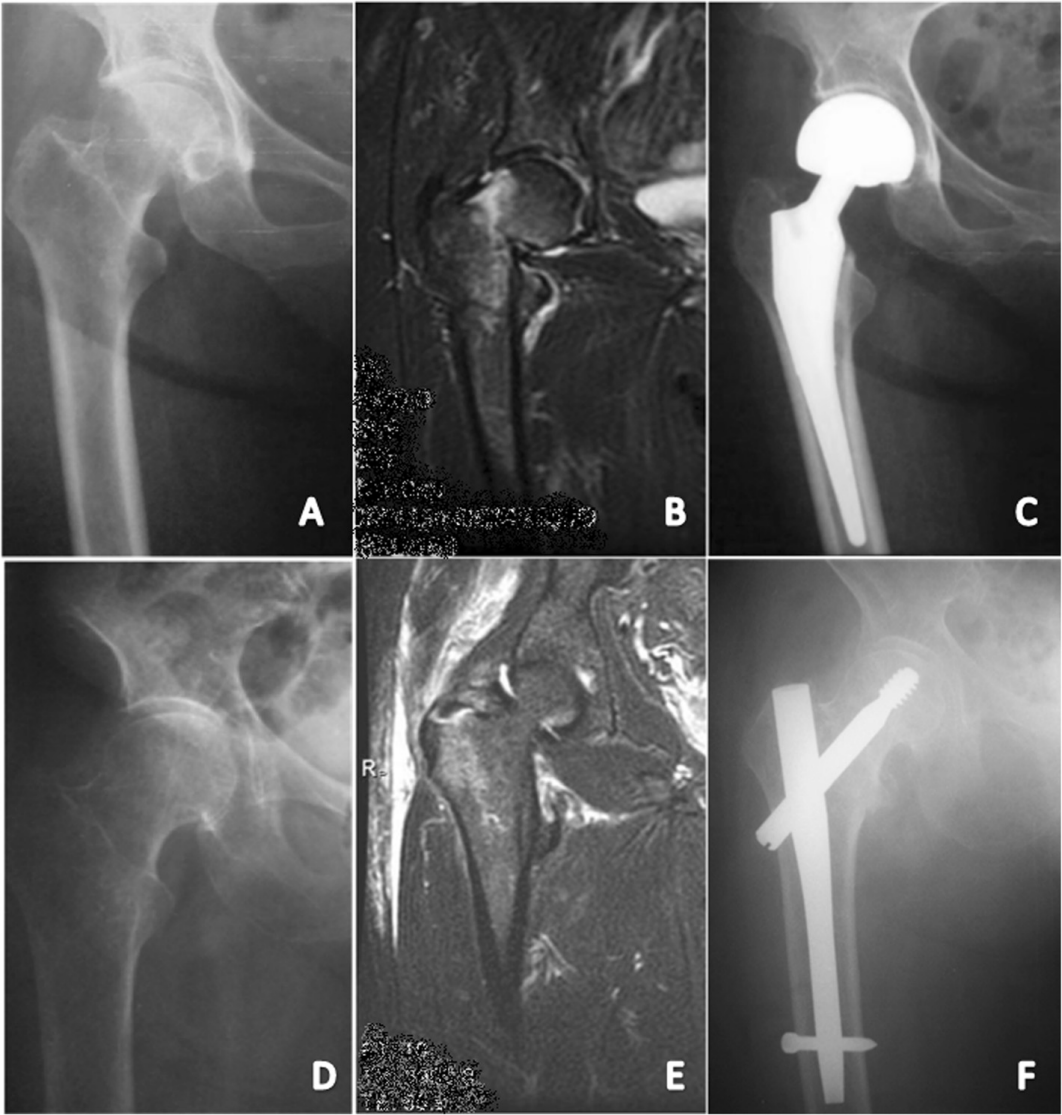
**a** X-Ray of occult femoral neck fracture of the right hip. **b** MRI of the same case showing a Garden II femoral neck fracture. **c** Postoperative X-ray showing a uncemented bipolar hip. **d** X-Ray of occult pertrochanteric fracture of the right hip. **e** MRI of the same case showing a incomplete pertrochanteric fracture (>50 %). **f** Postoperative X-ray showing osteosynthesis with a Gamma 3 nail (Stryker, NJ)

For femoral neck fractures in the elderly (Fig. 3a, b and c), the treatment of choice was hip hemiarthroplasty with a bipolar head and an uncemented stem (Taperlock, Biomet, Warsaw). Three parallel neck screws were used for 2 young patients with good bone stock. Pertrochanteric fractures (Fig. 3d, e and f) were routinely treated by intramedullary nailing (Gamma, Stryker, New Jersey) or extramedullary fixation with a sliding hip screw. Both techniques allowed immediate weight-bearing and early mobilization. Femoral neck fractures concomitant or iatrogenic were not detected in the patients undergoing nailing.

Out of the 82 cases investigated 34 were managed conservatively. Non-operative treatment consisted of bed rest initially and nonweightbearing on the affected leg no sooner than 3 weeks from the trauma. No attempt to manipulate the fracture was made since these were all nondisplaced fractures. No immobilization or skeletal traction was used. Progressive weightbearing was initiated at 6 weeks, starting with 10 %-20 % of total weight and increasing at a rate of 10 %-15 % per week under the direct supervision of a physical therapist. Radiographic evaluation was performed weekly for the first 4 weeks and monthly for the subsequent 3 months.

The relatively high rate of nonoperative treatment (34 cases, 46 % of total) was the result of a number of factors namely: incomplete fractures of the greater trochanter (4 cases, 4,9 %) and incomplete pertrochanteric fractures (13 cases, 15,8 %) In those cases the risk to benefit ratio would not justify the surgical intervention and the consensus of the trauma meeting of our units was in favor of conservative management. Furthermore associated comorbidities (7 cases, 8,5 %) were another rationale to selecting conservative treatment and finally the patient choice (10 cases, 12,2 %).

## Discussion

In the current study we present an imaging diagnosis protocol we have developed over the last 10 years using the best imaging modality available in an efficient manner and integrated this data with the selection of established treatment methods.

A hip MRI was preferred if available in the first 24 h. For all the 47 cases investigated by a primary MRI a positive occult hip fracture diagnosis was obtained. An emergency CT scan was performed in 35 cases when the MRI was not available during the first 24 h. This led to a true positive diagnosis in 12 cases. For the remaining 23 patients with negative x-rays and negative emergency CT, but with persistent hip pain, the diagnosis was obtained next day by an MRI scan in 14 cases, or a CT scan in 9 cases 72 h later.

If clinical suspicion of a missed fracture arises with inconclusive radiological results [14] different imaging studies should be employed to investigate the primary cause of hip pain [3]. Further radiographic investigation of the hip is usually not carried out with standardized (oblique, Judet, inlet, outlet) [4] or experimental views [15] or particular digital image processing techniques [16].

Both orthopedic surgeons and radiologists alike consider MRI to be the gold standard in the detection of occult fractures [3, 6, 17–22],with T(1)-weighted coronal MRI having 100 % sensitivity while for T(2)-weighted imaging there was 84.0 % sensitivity [23]. Pandey et al. conducted an ER based study evaluating patients with traumatic hip pain and negative X-rays by MRI. They identified 22 fractures out of 33 suspected cases, but did not provide a total number of hip fractures for the period [21]. Dominguez et al. found 4.4 % of patients with hip trauma and negative X-rays had a hip or pelvic fracture identified by MRI, representing 9.9 % of the total number of hip fractures [4].

CT scanning has been employed for the detection of occult hip fractures [24] with 93 % sensitivity and 95 % specificity. A 72 h delay is advised for further improving sensitivity [25]. In a study by Dunker et al. of 193 hips in elderly patients with negative or inconclusive radiographs, CT scans performed within 24 h after low hip trauma detected 41 femoral neck and 68 pertrochanteric fractures [26]. Jordan et al. in a recent paper recognized the main advantage of CT in its availability (usually 24/7 in a trauma center) and its smaller cost compared to MRI, in conformity with other authors, restating the role of CT in investigating occult hip fractures [2, 5]. A comparative study by Lubovsky et al. concluded that MRI was more accurate and provided less misdiagnosis than CT on a relatively small group of 13 patients [19] while a paper by Hakkarinen reports 4 patients out of 24 occult hip fractures that had a negative 64 slice CT scan and a positive MRI, concluding that while CT scanning is a useful tool in detecting occult hip fractures (18 fractures out of 24 were identified by CT only) false negative results are possible even with 64 slice CT while no fractures were missed by MRI [11]. One of the main shortcomings of computer tomography is the emission of X-rays and the rather high dose of weight dependent ionizing radiation that the patient would receive that it is doubled if a repeat 72 h CT is necessary [27].

Another imaging investigation described in literature is bone scintigraphy using technetium Tc 99 m polyphosphate, with a sensitivity of 98 % [28]. It is dependent on the timing of the examination, with a lower sensitivity and specificity than MRI. It is also less reliable in very old patients and those with circulatory disturbances [12]. Furthermore, the time necessary for a MRI acquisition is much shorter, with trauma protocols taking less than fifteen minutes to perform [1]. Alternative modalities have been evaluated for their value in diagnosing proximal femur fractures. Sonography was found to have 100 % sensibility and 65 % specificity compared to MRI. It could therefore be proposed as a screening tool for occult hip fractures in the absence of readily available MRI [29]. Auscultatory percussion technique is another useful method to assess patients who present with post-traumatic hip pain and normal radiographs [30].

Limitations include the retrospective design of our study and the use of a 16 slice CT scanner, not the most efficient for diagnosing occult hip fractures [11]. The investigation was however performed by a senior radiologist and the evaluation was done together with a senior orthopedic surgeon limiting the bias and false negative results.

## Conclusions

In conclusion, any patient with a suspected hip fracture (meaning a patient with significant posttraumatic hip pain, spontaneous, on palpation, mobilization or gait) and negative X-rays should receive further imaging exploration in the first 24 h. If MRI is readily available it should be preferred as it has better accuracy in detecting occult hip fractures. If for whatever reason an MRI cannot be performed in the first 24 h, an emergency CT scan should be used instead. This is often easier to accomplish, as CT is available round the clock in trauma centers and from our experience sufficient for establishing a diagnosis for the majority of patients. However in the case of a negative 24 h CT the patient should be further investigated. Again the MRI should be the investigation of choice, and if not available followed by a repeat CT after 72 h.

### Ethics approval and consent to participate

The present study was brought before and approved by our institution IRB.

## Abbreviations

AP: Antero-posterior
ER: Emergency room
CT: Computer tomography
ICD-10: International Statistical Classification of Diseases and Related Health Problems 10th Revision
MRI: Magnetic resonance imaging
X-ray: Radiography.
